# Supplementary material for: Long- and Short-Term Trends in Outpatient Attendance by Speciality in Japan: A Joinpoint Regression Analysis in the Context of the COVID-19 Pandemic
Source: Int J Environ Res Public Health. 2023 Dec 1;20(23):7133. doi: 10.3390/ijerph20237133 (PMC10705918; doi:10.3390/ijerph20237133)
Supplement: Supplementary file 1 [file ijerph-20-07133-s001.zip › ijerph-2673708-SI.pdf]

Table S1. Number of annual claims per 1,000 people by speciality from 2009 to 2021.

Speciality	2009	2010	2011	2012	2013	2014	2015	2016	2017	2018	2019	2020	2021
Internal medicine	2249.6	2253.7	2312.3	2362.0	2367.0	2380.8	2401.9	2454.3	2442.7	2495.3	2521.9	2307.5	2375.7
Paediatrics	316.7	314.1	325.2	328.4	314.7	320.4	322.8	334.5	321.9	326.3	332.7	251.2	278.5
Surgery	261.9	255.0	255.0	253.2	248.9	246.2	244.2	241.5	232.9	224.8	222.9	196.9	185.6
Orthopedics	474.1	487.8	503.0	521.2	533.7	549.2	561.0	576.6	592.4	601.7	616.9	586.6	624.0
Dermatology	408.2	422.1	436.6	447.5	455.4	458.4	473.2	486.0	496.3	500.0	514.6	513.9	516.9
Obstetrics & gynaecology	153.8	155.1	154.9	156.0	156.3	156.0	157.5	157.7	157.7	159.0	163.4	159.6	171.3
Ophthalmology	635.2	642.4	643.1	656.8	661.4	656.5	665.4	664.3	665.4	668.8	669.9	620.4	640.4
Otolaryngology	399.5	413.3	434.7	438.1	445.4	450.0	466.8	470.9	474.5	486.3	502.7	395.4	416.6
Dentistry	1467.2	1490.3	1528.8	1571.4	1609.8	1659.8	1707.7	1683.9	1708.3	1735.1	1802.2	1695.0	1809.3

Table S2. Cost per day by speciality and its percentage of change between 2020 and 2021 and the corresponding periods in 2019.

		Jan	Feb	Mar	Apr	May	Jun	Jul	Aug	Sep	Oct	Nov	Dec
Speciality	Year	N (% change)											
Internal medicine	2019	8583	8653	8593	8653	8895	8733	8632	8823	8793	8640	8537	8613
	2020	8942	8776	8956	9066	9530	9346	9223	9268	9261	8982	9201	9270

		(4.2)	(1.4)	(4.2)	(4.8)	(7.1)	(7.0)	(6.9)	(5.0)	(5.3)	(4.0)	(7.8)	(7.6)
	2021	9758	9531	9362	9376	9588	9280	9338	9620	9574	9379	9327	9406
		(13.7)	(10.1)	(9.0)	(8.4)	(7.8)	(6.3)	(8.2)	(9.0)	(8.9)	(8.6)	(9.3)	(9.2)
Paediatrics	2019	5806	5651	5534	5439	5548	5362	5321	5576	5486	5456	5436	5669
	2020	5938	5711	5780	5862	6248	6091	6019	6255	6208	5879	5958	6399
		(2.3)	(1.1)	(4.4)	(7.8)	(12.6)	(13.6)	(13.1)	(12.2)	(13.1)	(7.8)	(9.6)	(12.9)
	2021	7104	6895	6774	6869	7050	6959	7065	7393	7297	6777	6601	6741
		(22.3)	(22.0)	(22.4)	(26.3)	(27.1)	(29.8)	(32.8)	(32.6)	(33.0)	(24.2)	(21.4)	(18.9)
Surgery	2019	7125	7049	6952	7000	7112	6998	6951	7168	7108	7052	7020	7090
	2020	7334	7209	7260	7249	7386	7301	7309	7349	7348	7199	7324	7391
		(2.9)	(2.3)	(4.4)	(3.6)	(3.8)	(4.3)	(5.2)	(2.5)	(3.4)	(2.1)	(4.3)	(4.3)
	2021	7786	7607	7478	7455	7614	7395	7502	7777	7655	7530	7525	7610
		(9.3)	(7.9)	(7.6)	(6.5)	(7.1)	(5.7)	(7.9)	(8.5)	(7.7)	(6.8)	(7.2)	(7.3)
Orthopaedics	2019	4318	4196	4136	4200	4320	4175	4139	4314	4229	4228	4210	4273
	2020	4412	4294	4307	4440	4542	4406	4380	4464	4382	4273	4321	4365
		(2.2)	(2.3)	(4.1)	(5.7)	(5.1)	(5.5)	(5.8)	(3.5)	(3.6)	(1.1)	(2.6)	(2.2)
	2021	4570	4415	4353	4364	4473	4358	4430	4526	4430	4351	4352	4404
		(5.8)	(5.2)	(5.2)	(3.9)	(3.5)	(4.4)	(7.0)	(4.9)	(4.8)	(2.9)	(3.4)	(3.1)
Dermatology	2019	3958	3937	3938	3918	4007	3958	3916	3902	3905	3920	3926	3896
	2020	3977	3940	3932	3872	3968	3980	3928	3906	3910	3914	3901	3886
		(0.5)	(0.1)	(-0.1)	(-1.2)	(-1.0)	(0.6)	(0.3)	(0.1)	(0.1)	(-0.2)	(-0.6)	(-0.2)
	2021	3979	3984	4003	4019	4058	4046	4019	4008	4004	3952	3934	3914
		(0.5)	(1.2)	(1.7)	(2.6)	(1.3)	(2.2)	(2.6)	(2.7)	(2.5)	(0.8)	(0.2)	(0.5)

Obstetrics & gynaecology	2019	6284	6271	6219	6183	6289	6264	6205	6195	6247	6205	6211	6184
	2020	6362	6257	6305	6257	6431	6534	6478	6470	6491	6483	6466	6448
		(1.2)	(-0.2)	(1.4)	(1.2)	(2.3)	(4.3)	(4.4)	(4.4)	(3.9)	(4.5)	(4.1)	(4.3)
	2021	6663	6601	6570	6502	6568	6582	6534	6567	6574	6562	6542	6539
		(6.0)	(5.3)	(5.6)	(5.2)	(4.4)	(5.1)	(5.3)	(6.0)	(5.2)	(5.8)	(5.3)	(5.7)
Ophthalmology	2019	7830	7804	7466	7531	7693	7612	7767	7278	7574	8058	8014	7564
	2020	8136	7876	7881	8141	7956	8059	7939	7541	8022	8231	8226	7897
		(3.9)	(0.9)	(5.6)	(8.1)	(3.4)	(5.9)	(2.2)	(3.6)	(5.9)	(2.1)	(2.7)	(4.4)
	2021	8385	8213	8323	8174	8099	8249	8038	8016	8368	8467	8473	8076
		(7.1)	(5.2)	(11.5)	(8.5)	(5.3)	(8.4)	(3.5)	(10.1)	(10.5)	(5.1)	(5.7)	(6.8)
Otolaryngology	2019	4674	4600	4386	4297	4633	4565	4534	4602	4615	4556	4483	4487
	2020	4745	4585	4375	4272	4746	4928	4828	4862	4912	4791	4699	4741
		(1.5)	(-0.3)	(-0.2)	(-0.6)	(2.4)	(8.0)	(6.5)	(5.7)	(6.4)	(5.2)	(4.8)	(5.7)
	2021	5102	5058	4828	4912	5183	5187	5179	5305	5276	5120	4986	4922
		(9.2)	(10.0)	(10.1)	(14.3)	(11.9)	(13.6)	(14.2)	(15.3)	(14.3)	(12.4)	(11.2)	(9.7)
Dentistry	2019	6902	7086	7048	7051	7008	7088	7043	7061	7107	7191	7198	7269
	2020	7040	7214	7229	7580	7546	7582	7621	7618	7664	7588	7575	7674
		(2.0)	(1.8)	(2.6)	(7.5)	(7.7)	(7.0)	(8.2)	(7.9)	(7.8)	(5.5)	(5.2)	(5.6)
	2021	7461	7669	7663	7728	7721	7769	7749	7747	7783	7809	7751	7861
		(8.1)	(8.2)	(8.7)	(9.6)	(10.2)	(9.6)	(10.0)	(9.7)	(9.5)	(8.6)	(7.7)	(8.1)

Table S3. Days per claim by speciality and its percentage of change between 2020 and 2021 and the corresponding periods in 2019.

Speciality	Year	Jan<br>N (% change)	Feb	Mar	Apr	May	Jun	Jul	Aug	Sep	Oct	Nov	Dec
Internal medicine	2019	1.405	1.403	1.448	1.465	1.431	1.445	1.489	1.452	1.442	1.471	1.442	1.431
	2020	1.397	1.398	1.428	1.428	1.417	1.446	1.453	1.421	1.437	1.459	1.399	1.423
		(-0.6)	(-0.3)	(-1.4)	(-2.5)	(-1.0)	(0.0)	(-2.4)	(-2.2)	(-0.3)	(-0.8)	(-2.9)	(-0.5)
	2021	1.376	1.371	1.439	1.439	1.398	1.436	1.436	1.409	1.416	1.421	1.407	1.405
		(-2.1)	(-2.3)	(-0.7)	(-1.8)	(-2.3)	(-0.6)	(-3.5)	(-3.0)	(-1.8)	(-3.4)	(-2.4)	(-1.8)
Paediatrics	2019	1.469	1.456	1.517	1.551	1.493	1.552	1.564	1.452	1.515	1.524	1.517	1.523
	2020	1.438	1.475	1.454	1.416	1.331	1.382	1.414	1.367	1.391	1.486	1.434	1.451
		(-2.1)	(1.3)	(-4.2)	(-8.7)	(-10.9)	(-10.9)	(-9.6)	(-5.9)	(-8.2)	(-2.5)	(-5.5)	(-4.7)
	2021	1.351	1.360	1.433	1.493	1.465	1.535	1.566	1.437	1.408	1.450	1.469	1.459
		(-8.0)	(-6.6)	(-5.6)	(-3.7)	(-1.9)	(-1.1)	(0.1)	(-1.0)	(-7.1)	(-4.8)	(-3.2)	(-4.2)
Surgery	2019	1.718	1.745	1.824	1.838	1.788	1.829	1.882	1.789	1.801	1.837	1.794	1.766
	2020	1.709	1.721	1.777	1.751	1.740	1.808	1.798	1.734	1.771	1.821	1.730	1.750
		(-0.5)	(-1.3)	(-2.6)	(-4.7)	(-2.7)	(-1.1)	(-4.5)	(-3.1)	(-1.7)	(-0.9)	(-3.5)	(-0.9)
	2021	1.664	1.685	1.796	1.796	1.713	1.778	1.755	1.693	1.732	1.757	1.724	1.714
		(-3.1)	(-3.4)	(-1.5)	(-2.3)	(-4.2)	(-2.8)	(-6.8)	(-5.3)	(-3.9)	(-4.4)	(-3.9)	(-2.9)
Orthopaedics	2019	2.532	2.605	2.765	2.728	2.594	2.707	2.812	2.540	2.635	2.718	2.687	2.638

	2020	2.531	2.589	2.691	2.544	2.516	2.702	2.658	2.468	2.598	2.751	2.587	2.609
		(0.0)	(-0.6)	(-2.7)	(-6.8)	(-3.0)	(-0.2)	(-5.5)	(-2.8)	(-1.4)	(1.2)	(-3.7)	(-1.1)
	2021	2.436	2.522	2.744	2.692	2.495	2.656	2.574	2.450	2.588	2.659	2.617	2.566
		(-3.8)	(-3.2)	(-0.8)	(-1.3)	(-3.8)	(-1.9)	(-8.5)	(-3.6)	(-1.8)	(-2.2)	(-2.6)	(-2.7)
Dermatology	2019	1.275	1.284	1.321	1.314	1.272	1.306	1.328	1.279	1.299	1.317	1.301	1.290
	2020	1.278	1.289	1.310	1.299	1.283	1.315	1.323	1.274	1.307	1.328	1.288	1.284
		(0.3)	(0.4)	(-0.8)	(-1.1)	(0.9)	(0.7)	(-0.4)	(-0.4)	(0.6)	(0.8)	(-1.0)	(-0.4)
	2021	1.266	1.277	1.318	1.312	1.279	1.308	1.308	1.274	1.300	1.309	1.299	1.283
		(-0.7)	(-0.6)	(-0.2)	(-0.2)	(0.6)	(0.2)	(-1.5)	(-0.4)	(0.1)	(-0.6)	(-0.2)	(-0.5)
Obstetrics & gynaecology	2019	1.449	1.448	1.498	1.494	1.469	1.486	1.516	1.466	1.469	1.490	1.470	1.460
	2020	1.436	1.450	1.474	1.448	1.445	1.474	1.479	1.439	1.455	1.480	1.430	1.444
		(-0.9)	(0.2)	(-1.6)	(-3.1)	(-1.6)	(-0.8)	(-2.5)	(-1.9)	(-1.0)	(-0.7)	(-2.7)	(-1.1)
	2021	1.407	1.412	1.465	1.458	1.423	1.451	1.449	1.410	1.421	1.436	1.423	1.414
		(-2.9)	(-2.4)	(-2.2)	(-2.4)	(-3.1)	(-2.4)	(-4.5)	(-3.9)	(-3.3)	(-3.6)	(-3.2)	(-3.2)
Ophthalmology	2019	1.163	1.167	1.180	1.179	1.159	1.173	1.189	1.160	1.166	1.184	1.183	1.170
	2020	1.166	1.171	1.186	1.189	1.161	1.173	1.177	1.153	1.167	1.185	1.172	1.171
		(0.2)	(0.4)	(0.4)	(0.9)	(0.2)	(0.0)	(-1.0)	(-0.6)	(0.1)	(0.1)	(-0.9)	(0.1)
	2021	1.162	1.160	1.183	1.183	1.156	1.173	1.173	1.154	1.169	1.179	1.175	1.170
		(-0.2)	(-0.6)	(0.2)	(0.3)	(-0.3)	(0.0)	(-1.4)	(-0.5)	(0.2)	(-0.4)	(-0.6)	(0.0)
Otolaryngology	2019	1.477	1.412	1.456	1.487	1.454	1.508	1.547	1.464	1.492	1.504	1.518	1.532
	2020	1.455	1.410	1.424	1.408	1.388	1.449	1.467	1.408	1.428	1.473	1.451	1.482
		(-1.5)	(-0.1)	(-2.2)	(-5.3)	(-4.5)	(-4.0)	(-5.2)	(-3.8)	(-4.2)	(-2.1)	(-4.4)	(-3.3)

	2021	1.388	1.309	1.377	1.420	1.401	1.453	1.470	1.404	1.415	1.434	1.446	1.445
		(-6.1)	(-7.3)	(-5.4)	(-4.5)	(-3.6)	(-3.6)	(-4.9)	(-4.1)	(-5.1)	(-4.7)	(-4.7)	(-5.7)
Dentistry	2019	1.718	1.734	1.777	1.766	1.720	1.736	1.792	1.668	1.710	1.749	1.736	1.696
	2020	1.691	1.707	1.774	1.815	1.769	1.801	1.778	1.675	1.709	1.761	1.688	1.677
		(-1.5)	(-1.6)	(-0.1)	(2.8)	(2.9)	(3.7)	(-0.8)	(0.4)	(-0.1)	(0.6)	(-2.7)	(-1.1)
	2021	1.658	1.668	1.733	1.715	1.652	1.698	1.672	1.616	1.657	1.694	1.676	1.628
		(-3.5)	(-3.8)	(-2.5)	(-2.9)	(-4.0)	(-2.2)	(-6.7)	(-3.1)	(-3.1)	(-3.2)	(-3.4)	(-4.0)
